# Development of schizogenous intercellular spaces in plants

**DOI:** 10.3389/fpls.2015.00497

**Published:** 2015-07-02

**Authors:** Kimitsune Ishizaki

**Affiliations:** Graduate School of Science, Kobe University, Kobe, Japan

**Keywords:** aerenchyma, cell separation, cell wall remodeling, extracellular signaling, intercellular space formation, *Marchantia polymorpha*

## Abstract

Gas exchange is essential for multicellular organisms. In contrast to the circulatory systems of animals, land plants have tissues with intercellular spaces (ICSs), called aerenchyma, that are critical for efficient gas exchange. Plants form ICSs by two different mechanisms: schizogeny, where localized cell separation creates spaces; and lysogeny, where cells die to create ICSs. In schizogenous ICS formation, specific molecular mechanisms regulate the sites of cell separation and coordinate extensive reorganization of cell walls. Emerging evidence suggests the involvement of extracellular signaling, mediated by peptide ligands and leucine-rich repeat receptor-like kinases, in the regulation of cell wall remodeling during cell separation. Recent work on the liverwort *Marchantia polymorpha* has demonstrated a critical role for a plasma membrane-associated plant U-box E3 ubiquitin ligase in ICS formation. In this review, I discuss the mechanism of schizogenous ICS formation, focusing on the potential role of extracellular signaling in the regulation of cell separation.

## Introduction

Gas exchange is vital for all living organisms. Single-celled or small multicellular organisms carry out gas exchange via diffusion directly between cells and the environment or through the gastrovascular cavity. Complex multicellular organisms achieve gas exchange through specialized systems for gas uptake and circulation. For example, vertebrates have a closed circulatory system in which blood is pumped by a heart, and gas exchange occurs through lungs or gills. Although plants lack a circulatory system, many develop intercellular spaces (ICSs), which are connected directly to the external environment and provide efficient gas exchange via stomata or air pores (Raven, 1996; Jackson and Armstrong, 1999; Evans, 2004). Development of ICSs for gas exchange is critical for photosynthesis and transpiration in plants because the diffusion coefficients of carbon dioxide and oxygen are 10,000 times lower in water than in air (Voesenek et al., 2006). In the leaves of vascular plants, spongy mesophyll is a typical example of tissue containing ICSs for efficient gas exchange. As a result of ICS formation in mesophyll, the internal surface area of a leaf may be between 7 and 32 times larger than its external surface area, depending on the species and on environmental factors such as light intensity or quality (Dale, 1988).

Intercellular spaces are common and well developed in the roots and stems of wetland plants that grow in hypoxic soils (Seago et al., 2005; Joshi and Kumar, 2012). ICSs are also observed in bryophytes, such as the air chambers in the gametophyte of some liverworts, and sporophyte substomatal cavities of mosses and hornworts (Renzaglia et al., 2000). ICSs and stomata are recorded in fossils of early land plants found in the Rhynie chert, which is approximately 400–412 million years old, indicating that ICSs are among the most conservative characters in the evolution of land plants (Edwards et al., 1998). In addition to the evolution of stomata, the cuticle, lignified cell walls, and the water conduction system, internalization of the gas exchange surface by ICSs is likely one of the most critical land plant adaptations to terrestrial environments (Raven, 2002).

Intercellular spaces in plants are formed either via lysogeny or schizogeny. A lysigenous ICS is caused by spatially specified cell death to leave a space. Lysigenous ICS formation occurs in the roots of many crop species, including rice (Justin and Armstrong, 1991), maize (He et al., 1996; Gunawardena et al., 2001), and wheat (Trought and Drew, 1980). In contrast, schizogenous ICSs are formed by cell separation, instead of cell death. Schizogenous ICS formation is common in the roots and stems of wetland plants, such as species of *Rumex* and *Sagittaria*. In most vascular plants, ICSs in the leaf mesophyll are schizogenous, formed by the partial separation of cells following the breakdown of cell wall components (Sachs et al., 1882; Dale, 1988). Schizogenous ICSs may be formed by differential growth, resulting in separation of adjacent cells from one another at the middle lamella of the cell walls. This process involves the differentiation of specialized cells that undergo cell wall breakdown, then subsequently divide and enlarge differentially, to generate ICSs by cell separation. Although the process of schizogenous ICS formation has been described at the histological level in some species, its developmental regulation and molecular mechanisms remain largely unknown (Jackson and Armstrong, 1999; Evans, 2004).

## Cell Wall Remodeling in Cell Separation

Cell separation is a critical aspect of various developmental processes, including the emergence of lateral roots, the shedding of damaged or senescent organs, the release of pollen from anthers and seeds from pods, the softening of fruits, and the formation of ICSs in leaf mesophyll or in the aerenchyma of waterlogged roots. The process of cell separation involves the breakdown of cell walls in a defined area of tissue (Patterson, 2001). ICS formation appears to require specialized modifications of the cell wall in most reported cases (Knox, 1992). In pea root tissues, the initial stage of ICS formation is preceded by the formation of a defined cell separation layer to open the gap (Roland, 1978). In bean leaves, ICS formation occurs schizogenously at newly formed cell junctions and involves highly localized breakdown of the cell wall in the parent cells, which continues in the region of the middle lamella of the parent and daughter cells (Jeffree et al., 1986). In pea cotyledons, the extent of separation at cell junctions is associated with localized wall thickenings and electron-dense structures (Kollöffel and Linssen, 1984). The localized lysis of cell wall components initiates the development of extensive ICSs in maize leaf mesophyll. Subsequently, these gaps are extended by the mechanical stresses exerted by distinctive cell shapes. This process is accompanied by localized wall thickening (Apostolakos et al., 1991).

The process of organ abscission has been studied in diverse angiosperm species, including rice, tomato, and *Arabidopsis thaliana*. It provides a model for well-organized cell separation, which involves breakdown of cell wall material between adjacent cells (Patterson, 2001). In organ abscission, cell separation is restricted to the narrow band of cells that compose the abscission layer (Aalen et al., 2013). In the first stage of organ abscission, cells in the abscission zone (AZ) differentiate to form small, isodiametric, and cytoplasmically dense cells. The differentiation of AZ cells occurs simultaneously with the development of lateral organs from the apical meristem (Sexton and Roberts, 1982; Roberts et al., 2002). The second stage is activation of abscission, in which the AZ cells become capable of responding to abscission signals, and the organs prepare to separate from the plant. Plant hormones are the major endogenous regulators of this process. In general, ethylene and jasmonate act as signals to accelerate abscission, whereas auxin, gibberellins, and brassinosteroids inhibit the process (Estornell et al., 2013). After the activation of abscission, pectic polysaccharides are broken down in the AZ cell walls, followed by cell expansion in the AZ layer. Various enzymes act on structural polysaccharides, leading to hydrolysis of the middle lamella and cell walls of the AZ cells. These enzymes include expansins, xyloglucan endotransglucosylase/hydrolases, *β*-1,4-glucanases (cellulases), and polygalacturonases (PGs; Estornell et al., 2013). Activation of the molecular machinery of abscission involves regulating the expression of numerous gene families that encode cell-wall-remodeling enzymes (Cai and Lashbrook, 2008; Ogawa et al., 2009; Estornell et al., 2013). Mutational analyses indicate the significance of PGs in cell separation events, including abscission (Ogawa et al., 2009). Following the actual separation, lignification of the abscission wound occurs to protect the plant from pathogen attack.

## Peptide Ligand and Receptor-Like Kinase in the Regulation of Cell Separation

The key genetic components that regulate cell separation have been studied using floral organ abscission mutants in *A. thaliana*. In wild-type flowers, the petals, sepals, and filaments abscise shortly after pollination or anthesis. The gene *INFLORESCENCE DEFICIENT IN ABSCISSION* (*IDA*), which encodes a short protein with an N-terminal signal peptide, has been identified as the causal gene of a mutant phenotype that causes flowers to be retained on a plant indefinitely (Butenko et al., 2003). The loss of function in genes encoding the leucine-rich repeat receptor-like kinase (RLK) HAESA (HAE) and its closely related, redundant partner HAESA-Like2 (HSL2) is sufficient to block organ abscission (Jinn et al., 2000; Cho et al., 2008). Morphological studies of floral organs show that specification of AZ cells is not affected in either the *ida* or *hae hsl2* double mutant. This finding suggests that both *IDA* and *HAE/HSL2* are expressed after the specification of AZ cells, and that they promote the activation of coordinated cell-wall-remodeling enzymes in AZ cells, which leads to cell separation. Considerable genetic and biochemical evidence supports a ligand–receptor relationship between IDA and HAE/HSL2 (Stenvik et al., 2006; Cho et al., 2008; Butenko et al., 2014). Several members of a mitogen-activated kinase cascade, namely, MITOGEN-ACTIVATED PROTEIN KINASE KINASE4 (MKK4), MKK5, MITOGEN-ACTIVATED PROTEIN KINASE3 (MAPK3) and MAPK6, act downstream of IDA and HAE/HSL2 signaling (Cho et al., 2008). The MADS-domain transcription factor AGAMOUS-like 15 (AGL15), which regulates expression of *HAE* through direct binding to the *HAE* promoter, is a direct target of MAPK phosphorylation. Phosphorylation by MAPK relieves AGL15 repression of *HAE* expression, leading to an increase in HAE receptor-mediated signaling, and thus completes a positive feedback loop controlling floral organ abscission (Patharkar and Walker, 2015). Overexpression of IDA confers early abscission, and production of a white substance in the floral AZs. The main components of the white substance are the cell wall monosaccharides arabinose and galactose, suggesting that IDA acts as a positive regulator of cell separation by promoting cell wall breakdown in the AZ (Stenvik et al., 2006; Cho et al., 2008). These effects of IDA overexpression are reversed when HAE and HSL2 activity is compromised (Stenvik et al., 2006; Cho et al., 2008). Microarray data suggest that the IDAHAE/HSL2 signaling module is involved in the regulation of cell-wall-remodeling genes (Cai and Lashbrook, 2008). Reduced transcription of cell-wall-modifying enzymes is observed in AZs of the *hae hsl2* mutant (Cho et al., 2008). Additionally, the IDA-HAE/HSL2 signaling module regulates expression of cell-wall-remodeling genes, which promotes degradation of pectic polysaccharides during the cell separation process of lateral root emergence (Kumpf et al., 2013). These findings indicate that the IDA-HAE/HSL2 signaling module has been adapted to function in different root and shoot cell-separation processes.

## An E3 Ubiquitin Ligase Regulates Cell Separation in ICS Formation

**FIGURE 1:**
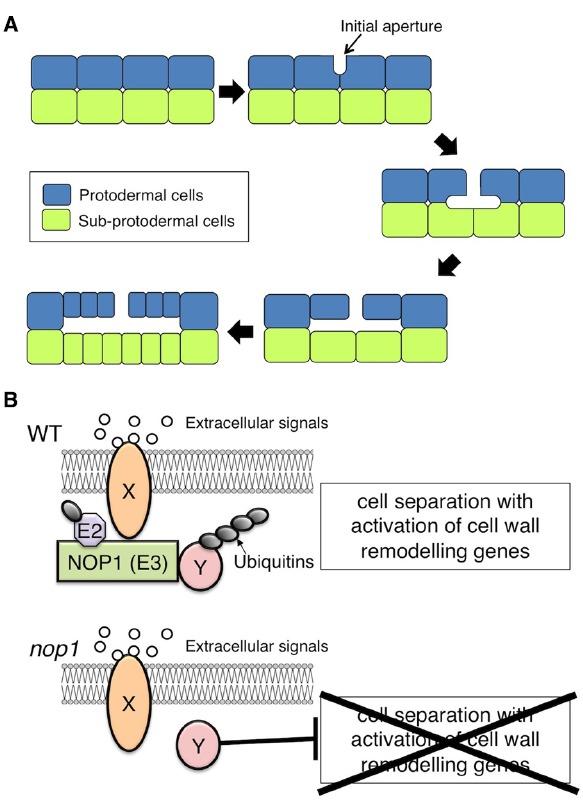
**Schizogenous intercellular space (ICS) formation in *Marchantia polymorpha.* (A)** Schematic representation of ICS formation during air chamber development. After a periclinal cell division to generate the protodermal and sub-protodermal cell layers, ICSs first appear as an initial aperture, between the anticlinal walls of protodermal cells. The base of the initial aperture broadens. The primary air chamber is formed via anticlinal cell divisions and protodermal and subprotodermal cell growth surrounding the ICS. **(B)** Hypothetical model of NOP1-mediated signaling involved in schizogenous ICS formation. Extracellular signals such as peptide ligands that are perceived by a receptor, Y, promote ICS formation via modulation of regulatory factor X that otherwise suppresses activation of cell-wall-remodeling enzymes. In the modulation of regulatory factor X, receptor Y signaling activates the E3 ligase NOP1 via phosphorylation of the ARM-repeat domain. NOP1 catalyzes the ubiquitination and subsequent degradation of regulatory factor X, which promotes cell separation through transcriptional activation of cell-wall-remodeling genes. In *nop1*, cell separation is constitutively suppressed by the action of regulatory factor X.

The gametophyte thallus of the liverwort *Marchantia polymorpha* contains a multilayered tissue with air chambers on the dorsal surface (Smith, 1955). The air chambers have an ICS that contains chloroplast-rich filaments developed from the subepidermis, and the ICS is connected directly to the external atmosphere through air pores in the epidermis of the chamber (Barnes and Land, 1907; Apostolakos et al., 1982). The development of the air pores and air chambers of *Marchantia* starts with the formation of schizogenous ICSs, termed “initial apertures,” between the anticlinal walls of protodermal cells in the apical region of the thallus (Apostolakos et al., 1982). ICS expansion from the initial apertures, which results from cell division and protodermal and subprotodermal cell growth around the initial aperture, leads to the formation of an air chamber (Figure 1A; Apostolakos et al., 1982).

For more than a century, *M. polymorpha* has been the subject of developmental and physiological studies. It is regarded as an emerging model organism because of its ease of growth, basal evolutionary position among land plants, simple genetic architecture and genetic transformability (Ishizaki et al., 2008; Kubota et al., 2013), and the ease of gene-targeting (Ishizaki et al., 2013a; Sugano et al., 2014). Using an efficient *Agrobacterium*-mediated transformation protocol (Ishizaki et al., 2008), a mutant impaired in air chamber formation, *nopperabo1* (*nop1*), was isolated from 10,000 T-DNA-tagged lines (Ishizaki et al., 2013b). In *nop1*, cell wall separation during initial aperture formation is impaired. The causal gene of *nop1* was identified as *NOPPERABO1* (*NOP1*), which encodes a Plant U-box (PUB) protein that carries tandem ARMADILLO (ARM) repeats in the C-terminus and localizes on the plasma membrane (Ishizaki et al., 2013b).

The U-box domain is similar in structure to the RING finger motif. U-box proteins function as E3 ubiquitin ligases and catalyze ubiquitin transfer from the ubiquitin-conjugating enzyme (E2) to the target for ubiquitination (Aravind and Koonin, 2000; Ohi et al., 2003). PUB proteins contain a variety of domain organizations and, in many cases, carry additional predicted domains, such as serine/threonine kinase, tetratricopeptide repeat, WD40 repeat, and ARM repeat domains. The ARM repeat domain is a highly conserved right-handed superhelix of *α*-helices involved in protein–protein interactions (Samuel et al., 2006; Tewari et al., 2010). Thus, the location of NOP1 in plasma membrane may depend on its C-terminus ARM repeats, via their interaction with regulatory proteins located on or near the plasma membrane.

## Model of NOP1 Function in Schizogenous ICS Formation

Initiation of ICS in *Marchantia* occurs at the corners of three or more protodermal cells located only in the apical notch region (Apostolakos et al., 1982). To specify the position of cell wall remodeling for ICS formation, intercellular communication may participate in the mechanism to determine the relative position of the protodermal cells within the apical notch region.

Recent evidences point to connections between PUB-ARM proteins and RLK, and suggests a role for PUB-ARM proteins as potential signaling proteins for RLKs (Gu et al., 1998; Kim et al., 2003; Samuel et al., 2006, 2008; Mbengue et al., 2010; Lu et al., 2011). For example, in *A. thaliana*, the PUB-ARM proteins PUB12 and PUB13 diminish the activity of leucine-rich repeat RLK FLAGELLIN-SENSING 2 by ubiquitination and subsequent degradation (Lu et al., 2011). In *Brassica* and *Arabidopsis*, *S*-receptor kinase phosphorylates the ARM-repeat domain of the PUB-ARM protein ARK1, which acts as a positive regulator of the self-incompatibility response (Gu et al., 1998; Samuel et al., 2008).

NOP1 may be involved in a plasma membrane-localized RLK signaling pathway that regulates ICS formation in air chambers (Figure 1B). Because loss-of-function mutation of *NOP1* confers impaired cell wall separation, NOP1 could catalyze ubiquitination and subsequent degradation of the regulatory factor(s) that suppress cell wall separation (Figure 1B). Identification of NOP1-associated proteins will be crucial to understanding the molecular mechanisms of ICS formation.

As discussed above, the IDA-HAE/HSL2 signaling module regulates cell separation through transcriptional activation of cell-wall-remodeling genes, such as expansins, xyloglucan endotransglucosylase/hydrolases, cellulases, and *PGs* in *A. thaliana* (Cai and Lashbrook, 2008; Cho et al., 2008; Kumpf et al., 2013). In the initial process of air chamber formation of *M. polymorpha*, a local thickening and subsequent detachment of the cell wall occurs to form an initial aperture at the junction of three to five protodermal cells (Apostolakos and Galatis, 1985). The PUB-ARM E3-ligase NOP1 is involved in this process (Ishizaki et al., 2013b). Bryophytes show some distinct features in terms of cell wall composition compared with vascular plants, i.e., the lack of a lignified secondary cell wall and a decreased amount of borate cross-linked rhamnogalacturonan II in the primary cell wall. However, the primary cell wall of bryophytes shares some common components with vascular plants, such as cellulose microfibrils, mannose-containing hemicelluloses, and xyloglucans (Matsunaga et al., 2004; Sarkar et al., 2009). Homologs of cell-wall-remodeling genes for expansins, xyloglucan endotransglucosylase/hydrolases, cellulases, and PGs have been identified in the moss *Physcomitrella patens* genome (Davison and Blaxter, 2005; Carey and Cosgrove, 2007; Eklöf and Brumer, 2010; McCarthy et al., 2014). To achieve well-organized ICS formation, NOP1 might be involved in RLK signaling in a similar manner to IDA-HAE/HSL2, which regulates the transcriptional activity of cell-wall-remodeling genes. Further comparative genomic studies are required to shed light on the genetic and biochemical processes of cell wall remodeling in ICS formation of *M. polymorpha*, and contribute to our understanding of the fundamental mechanism of the cell-wall-remodeling machinery and its evolution in land plants.

## Conclusion

The development of ICSs is considered a significant event in the evolution of land plants, but little is known about the molecular mechanisms involved. The process of schizogenous ICS formation involves well-organized cell wall remodeling and may share a common regulatory mechanism with the process of organ abscission. In recent years, significant progress has been made in understanding many regulatory aspects of organ abscission, using the model plant *A. thaliana*. The extracellular signaling module that is mediated by peptide ligands and RLK molecules activates transcription of genes that encode cell-wall-remodeling enzymes, thus promoting organ abscission and the emergence of lateral roots. Knowledge of the mechanisms of organ abscission may be useful to design genetic and molecular strategies for understanding schizogenous ICS formation. Because *M. polymorpha* has a simple genome architecture, and is amenable both to transformation (Ishizaki et al., 2008; Kubota et al., 2013) and targeted genome modification (Ishizaki et al., 2013a; Sugano et al., 2014), it should be an effective model organism for studying ICS formation at the molecular level by examining air chamber development. Further investigations of NOP1-mediated air chamber development in *M. polymorpha* will contribute to our understanding of the fundamental regulatory mechanism and evolution of cell separation in land plants.

### Conflict of Interest Statement

The author declares that the research was conducted in the absence of any commercial or financial relationships that could be construed as a potential conflict of interest.
